# Wheat-derived arabinoxylan oligosaccharides with bifidogenic properties abolishes metabolic disorders induced by western diet in mice

**DOI:** 10.1038/s41387-018-0019-z

**Published:** 2018-03-07

**Authors:** Audrey M. Neyrinck, Sophie Hiel, Caroline Bouzin, Vicenta Garcia Campayo, Patrice D. Cani, Laure B. Bindels, Nathalie M. Delzenne

**Affiliations:** 1 Metabolism and Nutrition Research Group, Louvain Drug Research Institute, Université Catholique de Louvain, 1200 Brussels, Belgium; 2 IREC Imaging Platform (2IP), Institute of Experimental and Clinical Research, Université Catholique de Louvain, 1200 Brussels, Belgium; 3 Cargill R&D, Mineapolis, MN USA; 4 Walloon Excellence in Life sciences and BIOtechnology (WELBIO), Louvain Drug Research Institute, Université Catholique de Louvain, 1200 Brussels, Belgium

## Abstract

**Background:**

Non-digestible carbohydrates present in cereals such as fructans and arabinoxylans represent promising prebiotic nutrients to prevent the development of obesity and related metabolic disorders. OBJECTIVE AND DESIGN: The aim of this study was to determine the corrective effects of wheat bran-derived arabinoxylan oligosaccharides in obese mice fed a western diet (WD). WD was given for 4 weeks before wheat bran extract (WBE) supplementation (5%) for an additional 4 weeks, whereas a control group received the standard diet.

**Results:**

Bifidogenic effect of WBE was evidenced by an induction of both *Bifidobacterium animalis* and *Bifidobacterium pseudolongum* in the caecal content. WBE supplementation normalised WD-induced fat-mass expansion, steatosis, hypercholesterolemia, hyperleptinemia, hyperglycemia and hyperinsulinemia reaching the values of control mice. The reduced glucose-dependent insulinotropic polypeptide (GIP) release observed in WD + WBE mice may be a protective mechanism in terms of reducing adipose tissue storage, hepatic steatosis and glucose homoeostasis.

**Conclusion:**

We found that WBE completely abolished WD-induced metabolic disorders. Those results might be useful to take into account nutritional advices to treat obesity and related metabolic disorders such as type 2 diabetes, hypercholesterolaemia and fatty liver diseases when obesity was already established.

## Introduction

Obesity and related metabolic disorders such as diabetes, cardiovascular diseases and non-alcoholic fatty liver diseases become major health problems in Western countries, while the consumption of cereals has decreased markedly over the last century^1^. Dietary fibres are the most important constituents in cereal grains related to positive health effects, and the consumption of the whole grain products help to reach the recommended dietary fibre intake^2–4^. Wheat bran is a low-cost byproduct of conventional wheat milling. Hydrolysis products of arabinoxylan—named arabinoxylan oligosaccharides (AXOS)—are characterised by their average degree of arabinose substitution and their lower average degree of polymerisation^5^.

Gut microbiota is an important factor involved in the control of body weight and metabolic alterations occurring in obesity^6,7^. Several mechanisms are proposed, linking events occurring in the gut upon carbohydrate fermentation and the control of metabolic disorders^8^. We have previously shown that oligosaccharides issued from inulin-type fructans counteracted obesity and metabolic alteration development upon high-fat feeding including dyslipidemia and diabetes, by modulating the gut microbiota^9,10^. The glucagon-like peptide-1 (GLP-1), a satietogenic gut-derived peptide, contributes largely to explain those effects^9,11^. However, to date, no study has evaluated the potential effect of AXOS on metabolic disorders in a model of obesity pre-established upon western diet feeding, in relationship with gut microbiota modulation.

## METHODS

### Animals and diets

Twenty seven male C57BL6 mice (9 weeks old, Janvier laboratories, France) were housed in specific pathogen-free (SPF) conditions in groups of 3 mice per cage in a controlled environment (12-hour daylight cycle) with free access to food and water. After 1 week of acclimatisation, mice were divided into two groups: a control group (CT) fed with a control diet (low fat, no sucrose, matched, purified ingredient diet corresponding to D12450K, Ssniff, Germany; *n* = 9) and a group fed with a WD (high-fat diet corresponding to D12451, Ssniff, Germany; n = 18). The composition of the diets is presented in supplementary information. After 4 weeks of dietary treatment, WD-treated mice were divided into two subgroups fed a WD with or without 5% wheat bran-derived arabinoxylan oligosaccharides (WBE group). WBE contained 72% of AXOS with an average degree of polymerisation of 5 and a degree of substitution arabinose/xylose of 0.24 (Cargill R&D centre Europe BVBA, Belgium). Mice body weight, food intake and water intake were recorded twice a week. Total fat mass was determined using a 7.5 MHz Time domain-Nuclear magnetic resonance (LF50 minispec, Bruker, Germany). After a total of 8 weeks of dietary treatment and a 6-h period of fasting, mice were anesthetised with isoflurane (Forene®, Abbott, Queenborough, Kent, England) and blood samples were harvested. Mice were killed by cervical dislocation. Portal blood was taken in <30 s and directly flushed within tubes containing dipeptidyl peptidase IV inhibitor (Millipore, St Charles, MO, USA). Plasma was immediately collected after centrifugation and stored at −80°C. Liver, colon and caecum were carefully dissected, weighted and immersed in liquid nitrogen before storage at −80 °C; pieces of liver were embedded in OCT compound and frozen in nitrogen-cooled isopentane for histology. Mouse experiment was approved by and performed in accordance with the guidelines of the local ethics committee under the specific agreement numbers 2014/UCL/MD/022. Housing conditions were as specified by the Belgian Law of 29 May 2013, on the protection of laboratory animals (Agreement LA 1230314).

### Biochemical analysis

Hepatic content of lipids and plasma insulin, triglycerides, cholesterol and non-esterified fatty acid concentrations were measured as described in supplementary information. Portal concentrations of glucose-dependent insulinotropic peptide (GIP), glucagon-like peptide-1 (GLP-1), ghrelin and leptin were determined in 2 × 15 µl of plasma using a multiplex immunoassay kit (Bioplex, Bio-Rad) and measured using Luminex technology (Bioplex, Bio-Rad).

### Histochemical detection of liver lipids

Frozen liver sections were sliced at 5 µm, treated with oil red O and scanned as previously described^12^. The lipid area were determined on whole sections using the imaging software TissueIA (v 2.0.3, Leica Biosystems, Dublin, Ireland). Pixels corresponding to the oil red O staining were selected to create a colour profile. Total tissue area was defined by setting the tissue intensity threshold at 210 (grey value). Results were expressed as stained area (below threshold)/tissue area (below threshold). Two representative tissue pieces were analysed for each mice.

### Analysis of the gut bacteria

At the end of the experiment, the total caecum content was collected and weighed before storage at −80°C. Genomic DNA was extracted from the caecal content using a QIAamp DNA Stool Mini Kit (Qiagen, Germany). Absolute quantification of bacteria was performed as described in supplementary information.

### Statistical analysis

Data are presented as mean ± SEM. Statistical significance between groups was assessed by one-way ANOVA (two-way ANOVA for body weight evolution). The ANOVA tests were followed by post-hoc Tukey’s multiple comparison tests (post-hoc Bonferroni for body weight evolution) using GraphPad Prism software (San Diego, CA, USA).

## Results

**Fig. 1:**
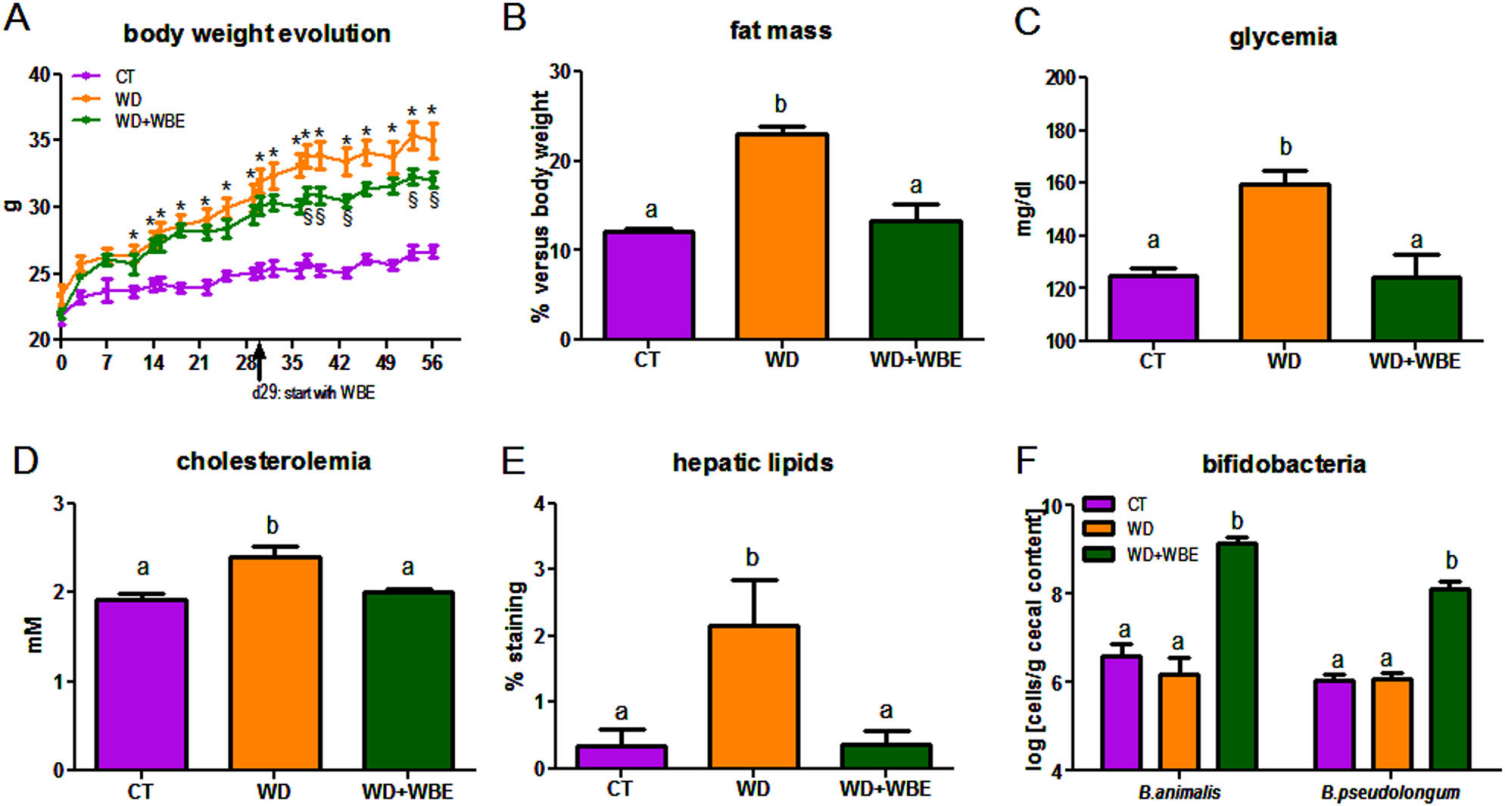
Prebiotic effect of wheat bran extract in the model of western diet-induced obesity and related metabolic alterations. Body weight evolution (**a**), fat mass measured by NMR at days 50 (**b**), glycemia (**c**), cholesterolemia (**d**), histological analysis of liver lipids (**e**) and bifidobacteria (**f**). Mice were fed a control diet (CT), a western diet (WD) or a WD supplemented with 5% of WBE (WD + WBE) for 4 weeks. Results are expressed as mean ± SEM. *p < 0.05 WD versus CT, ^§^*p* < 0.05 WD + WBE versus WD, data with different superscript letters are significantly different at *p* < 0.05 (ANOVA)

The WD consumption induced obesity after 4 weeks of dietary treatment (the body weight gain (d29-d0) was 2.91 ± 0.56 g and 6.97 ± 0.31 g for CT and WD groups, respectively; *p* < 0.05 Student *t-*test). WBE supplementation in the WD diet from d29 until d56 slowed down the body weight gain compared to the WD group (Fig. 1a) independently of the food intake (total food intake from d29 to d56: 70 ± 3, 64 ± 1, 70 ± 2 g for CT, WD and WD + WBE, respectively; *p* > 0.05, one-way ANOVA). Furthermore, WBE treatment blunted fat-mass expansion reaching the control value at the end of the treatment (Fig. 1b). The portal level of leptin, an adipokine produced proportionally to fat mass, was markedly increased in WD, whereas it decreased in WBE group (Table 1). In contrast, the plasma ghrelin was not significantly affected by the dietary treatments. Interestingly, WBE treatment counteracted the higher release of GIP induced by the WD. WBE supplementation upregulated proglucagon expression in the colon (1.00 ± 0.05 a, 1.05 ± 0.08 a and 1.33 ± 0.08 b for CT, WD and WD + WBE group, respectively; one-way ANOVA), whereas the plasma level of GLP-1 was not significantly affected (data not shown). WD-induced hyperglycemia, hyperinsulinemia, insulin resistance (HOMA-IR index) and hypercholesterolemia were decreased upon WBE supplementation, reaching the control values after 4 weeks of supplementation (Fig. 1c, d; Table 1). In contrast, plasma levels of triglycerides and free fatty acids were not significantly modified by the dietary treatments (Table 1). It is worth noting that higher hepatic content of lipids (mostly triglyceride content) observed upon WD was completely blunted by the WBE treatment (Fig. 1e). Finally, we determined the levels of several bacteria known to be affected by wheat bran product supplementation such as *Lactobacillus* spp., *Roseburia* spp., *Bacteroides* spp.^13,14^ but none of these bacteria was changed by the WBE supplementation (Table 1). We analysed two specific bifidobacteria species known to be present in mice and in humans: *Bifidobacterium animalis* and *Bifidobacterium pseudolongum*^15^. After only 4 weeks of WBE supplementation, we observed a marked bifidogenic effect for both species (Fig. 1f).

**Table 1:** Plasma parameters, liver lipids and caecal bacteria

	CT	WD	WD + WBE
Serum parameters			
–Triglycerides (mM)	0.36 ± 0.02	0.40 ± 0.02	0.40 ± 0.03
–Free fatty acids (mM)	0.40 ± 0.03	0.46 ± 0.03	0.48 ± 0.04
–Insulin (pg/ml)	0.15 ± 0.04^a^	0.75 ± 0.29^b^	0.13 ± 0.06^a^
–Leptin (pg/ml)	989 ± 112^a^	13967 ± 1583^b^	4647 ± 1370^a^
–Ghrelin (pg/ml)	122 ± 20	112 ± 26	76 ± 12
–GIP (pg/ml)	44 ± 5^a^	93 ± 12^b^	56 ± 8^a^
–HOMA-IR	9 ± 2^a^	60 ± 18^b^	15 ± 4^a^
Liver lipid content			
–Total lipids (mg/g tissue)	48 ± 2^a^	62 ± 4^b^	48 ± 4^a^
–Triglycerides (nmol/mg protein)	11.4 ± 0.9^a^	18.2 ± 2.5^b^	14.8 ± 1.4^ab^
–Cholesterol (nmol/mg protein)	8.5 ± 0.3^a^	11.9 ± 0.5^b^	11.3 ± 0.4^b^
Gut bacteria, log (cells/g caecal content)			
Total bacteria	11.71 ± 0.08	11.70 ± 0.06	11.60 ± 0.09
* Lactobacillus* spp.	8.46 ± 0.11	8.17 ± 0.17	8.41 ± 0.15
* Roseburia* spp.	8.97 ± 0.08	8.95 ± 0.08	8.91 ± 0.07
* Bacteroides* spp.	10.51 ± 0.12	10.33 ± 0.06	10.31 ± 0.13

Mice were fed a control diet (CT), a western diet (WD) or a WD supplemented with 5% of WBE (WD + WBE) for 4 weeks. Results are expressed as mean ± SEM. Data with different superscript letters are significantly different at *p* < 0.05 (ANOVA)

*GIP* glucose-dependent insulinotropic peptide, *HOMA-IR* homeostasis model assessment of insulin resistance

### Discussion and conclusion

We confirmed the prebiotic effect of AXOS produced from wheat bran in a model of WD-induced obesity. Indeed, WBE feeding for 4 weeks improved host physiology and this was associated with an increase in bifidobacteria demonstrated by an induction of two representative species in mice: *B. animalis* and *B. pseudolongum*. Previous studies reported that *B. animalis* is a specific gut bacterial strain negatively correlated with the body mass index that was able to reduce fat mass and glucose intolerance in both obese and diabetic mice when used a probiotic^16,17^.

This bifidogenic effect of WBE was associated with a normalisation of fat mass and plasma leptin due to a 8-week WD feeding supporting results obtained in our previous studies^12,14^. Interestingly, in our study this effect is independent of the caloric intake or the modulation of peptides regulating appetite such as ghrelin or GLP-1.

WD-induced type 2 diabetes, steatosis and hypercholesterolemia after 8 weeks of dietary treatment. Plasma levels of cholesterolemia, glycemia, insulinemia, the insulin resistance index (HOMA-IR) as well as the hepatic level of lipids returned to the levels obtained in control mice after only 4 weeks of WBE supplementation when obesity is already established. Therefore, this is the first study suggesting that wheat bran-derived AXOS is effective to counteract obesity instead of preventing it as previously shown elsewhere^12,14^. The lack of effect on triglyceridemia is not surprising as the serum triglycerides is dependent not only on VLDL secretion by the liver, but also on the lipoprotein lipase in other tissues. Importantly, the reduced GIP release observed in WD + WBE mice may be a protective mechanism in terms of reducing adipose tissue storage, hepatic steatosis and improving insulin sensitivity^18,19^.

In conclusion, this study reinforces the promising beneficial aspects of wheat bran-derived AXOS as prebiotic nutrient as it improved obesity-related metabolic disorders such as type 2 diabetes, hypercholesterolemia and fatty liver disease when obesity was already established.

## Electronic supplementary material

Supplementary information
