# Supplementary material for: Wheat-derived arabinoxylan oligosaccharides with bifidogenic properties abolishes metabolic disorders induced by western diet in mice
Source: Nutr Diabetes. 2018 Mar 7;8:15. doi: 10.1038/s41387-018-0019-z (PMC5856735; doi:10.1038/s41387-018-0019-z)
Supplement: Supplementary file 1 — Supplementary information [file 41387_2018_19_MOESM1_ESM.docx]

**Supplementary information**

Composition of the diets

|  |  | **CT** | **WD** |
| --- | --- | --- | --- |
| Protein | (kcal %) | 20 | 20 |
| Carbohydrate | (kcal %) | 70 | 35 |
| Fat | (kcal %) | 10 | 45 |
|  | kcal/gm | 3.85 | 4.73 |
| Casein | (%) | 20.7 | 25 |
| L-cystine | (%) | 0.25 | 0.3 |
| Corn Starch | (%) | 48.9 | 7 |
| Maltodextrin | (%) | 14 | 11 |
| Sucrose | (%) | 0 | 20.7 |
| Cellulose | (%) | 5 | 5.7 |
| Soybean oil | (%) | 2.4 | 2.8 |
| Lard | (%) | 1.6 | 20.8 |
| Mineral Mix | (%) | 6 | 6 |
| Vitamin Mix | (%) | 1 | 1 |
| Choline Chloride | (%) | 0.2 | 0.2 |

Biochemical analysis

Plasma insulin concentrations were determined using an ultrasensitive ELISA kit (Mercodia, Uppsala, Sweden). The Homeostasis Model Assessment of insulin resistance (HOMA-IR) was calculated by the following formula: HOMA-IR=[fasting glycemia (mM)]*[fasting insulinemia (pM)]/22.5 (1). Plasma triglycerides, cholesterol and free fatty acid concentrations were measured using kits coupling enzymatic reaction and spectrophotometric detection of reaction endproducts (Diasys Diagnostic and Systems, Holzheim, Germany). Lipid content was measured in the liver tissue after extraction with chloroform–methanol according to the Folch method. Briefly, 100 mg of liver were homogenised in 2 ml of chloroform: methanol (2:1). The chloroform phase was evaporated under nitrogen flux and the dried residue was weighted and solubilised in 1.5 ml of isopropanol. Triglyceride and cholesterol concentrations were measured using a kit coupling an enzymatic reaction and spectrophotometric detection of the final product (Diasys Diagnostic and System, Holzheim, Germany). Protein concentrations were measured by the Bradford method using bovine serum albumin as standard.

Quantitative polymerase chain reaction (qPCR) analysis of the 16S rRNA gene

q-PCR was performed with a StepOnePlus Real-Time PCR System and software (Applied Biosystems, Den Ijssel, The Netherlands) using Mesa Fast qPCR™ (Eurogentec, Seraing, Belgium) for detection. The primers are detailed in the following table:

| Total bacteria (Bacteria Universal 338F and 518R) | ACTCCTACGGGAGGCAGCAG | ATTACCGCGGCTGCTGG |
| --- | --- | --- |
| *Bifidobacterium pseudolongum* | CCCTTTTTCCGGGTCCTGT | ATCCGAACTGAGACCGGTT |
| *Bifidobacterium animalis* | ACCAACCTGCCCTGTGCACCG | CCATCACCCCGCCAACAAGCT |
| *Bacteroides* – *Prevotella* | GAGAGGAAGGTCCCCCAC | CGCTACTTGGCTGGTTCAG |
| *Lactobacillus* | AGCAGTAGGGAATCTTCCA | CACCGCTACACATGGAG |
| *Roseburia* | AAGCGACGATCAGTAGCCGA | TTCTTCTTCCCTGCTGATAGAG |

The cycle threshold of each sample was compared with a standard curve made by diluting genomic DNA isolated from a pure culture of a type strain (BCCM/LMG, Ghent, Belgium; DSMZ, Braunshweig, Germany). Thermal cycling consisted of an initial cycle of 95 °C 10 min, followed by 40 cycles of 95 °C 15 s, and 1 min at the appropriate primer-pair temperature. The bacterial concentration of each sample was calculated by comparing the Ct obtained from standard curves in which the Ct values were plotted as a linear function of the base-10 logarithm of the number of cells calculated by plate counting. Samples were analyzed in duplicate.

(1) Muniyappa R, Lee S, Chen H, Quon MJ. Current approaches for assessing insulin sensitivity and resistance in vivo: advantages, limitations, and appropriate usage. *Am J Physiol Endocrinol Metab* 2008; **294**: E15-E26
